# Clinical characteristics in new psychoactive substance users: A single center study

**DOI:** 10.1097/MD.0000000000034084

**Published:** 2023-06-23

**Authors:** Yu-Jang Su, Tse-Hao Chen, Wei-Hsiang Liao, Kuo-Song Chang, Yen-Chun Lai

**Affiliations:** a Toxicology Division, Department of Emergency Medicine, MacKay Memorial Hospital, Taipei, Taiwan; b Department of Emergency Medicine, MacKay Memorial Hospital, Taipei, Taiwan; c Department of Medicine, MacKay Medical College, New Taipei City, Taiwan; d MacKay Junior College of Medicine, Nursing, and Management, Taipei City, Taiwan; e Department of Nursing, Yuanpei University of Medical Technology, HsinChu, Taiwan; f Department of Emergency Medicine, TAMSUI BRANCH, Mackay Memorial Hospital, Taipei, Taiwan; g Department of Disaster Medicine, MacKay Memorial Hospital, Taipei, Taiwan; h Department of Anesthesiology. Taipei Medical University Hospital, Taipei, Taiwan.

**Keywords:** classical illicit drug, new psychoactive substance, poisoning

## Abstract

New psychoactive substances (NPS) are emerging illegal substances or synthetic drugs that pose public health threats worldwide. This study was aimed at reporting the clinical characteristics of NPS and classical illicit substances used by patients who presented to the emergency room. We conducted a retrospective cohort study on patients with suspected illicit substance use who visited the emergency department (ED) with the suspicion of illicit substance use. We divided the patients into 4 groups based on the NPS testing results: NPS positive, NPS negative, NPS combined with classical illicit drugs (INPS), and subjects with negative testing results. The majority of patients in all groups were male. The NPS users were significantly younger than those with negative results on toxic testing (26.4 vs 37.5, *P* = .005 < 0.05). The heart rate of NPS users was significantly faster than that of the group with negative results of toxic testing (111.1 vs 93.5 beats per minute, *P* = .046). The heartbeats of INPS group were also significantly faster than those with a negative result in toxicology screen (119.6 vs 93.5 beats per minute, *P* = .024). Those who used classical illicit drugs combined with NPS had significantly higher palpitation than those with negative results of toxic testing (27.3% vs 3.1%, *P* = .017). Patients who were highly suspicious of NPS use were younger, had tachycardia, felt palpitations, and had fair oxygen saturation compared to patients who were negative for urine toxicity screening.

## 1. Introduction

New psychoactive substances (NPS) are emerging illegal substances that pose a threat to public health worldwide. At the end of the 1990s, NPS mainly circulated among rave and electronic music parties in Europe.^[1]^ According to an Asian multicenter study, NPS users were 5.7 years younger than classical drug users, and over a quarter (27.4%) of school-going adolescents had tried 1 type of psychoactive substance at least once.^[2,3]^ NPS are either designed or synthetic drugs created to mimic the psychoactive effects of classical illicit drugs. Approximately 1004 individual NPS have been reported to the United Nations Office on Drugs and Crime in 125 countries and territories.^[4]^ Concurrently, NPS have been categorized into 6 groups: stimulants, synthetic cannabinoids, classic hallucinogens, synthetic opioids, hypnotics, and dissociatives. The detection of NPS requires high-sensitivity gas chromatography-mass spectrometry and ultra-high-performance liquid chromatography-high resolution mass spectrometry rather than a kit-based urine test.^[5,6]^

Cannabis is the most widely used drug worldwide, either as a classical illicit drug or a therapeutic agent that provides positive effects such as pain control, relaxation, and euphoria. Synthetic cannabinoids can mimic cannabis-like effects and cannot be detected by a simple urine toxicity test. Synthetic cannabinoids are the most diverse group of NPS, with many different structures and forms. They are more potent cannabinoid receptor agonists than traditional cannabis. Substances such as benzodiazepines and active metabolites of opioids are often added to synthetic cannabinoids, resulting in a wide range of unpredictable adverse effects.^[7]^ The symptoms of synthetic cannabinoid drug intoxication include nausea, agitation, diaphoresis, palpitations, hypertension, and seizures. Acute psychotic disorders, such as cognitive impairment and schizophrenia, have been reported.^[8]^

Synthetic stimulants are the most common type of NPS, and they can mimic the effects of established substances with stimulant properties, such as cocaine and amphetamines. These agents may cause sympathomimetic toxicity, such as agitation, nausea, headache, palpitations, hypertension, chest pain, hyperthermia, and psychoactive symptoms, such as agitation, hallucinations, and altered consciousness. Less common adverse effects, such as seizures, rhabdomyolysis, and cardiac arrest, have been reported.^[9]^ In 2017, mephedrone, a synthetic cathinone, was the predominant NPS used in Taiwan.^[10]^ The characteristics of these synthetic NPS are quite variable in fatal doses, such as low concentrations of synthetic cannabinoids or opioids. In contrast, the toxic concentrations of amphetamine-like derivatives are relatively higher.^[11]^ In an Italian study, NPS was also predictive of binge drinking behavior.^[12]^

Classical illicit drugs such as opioids, amphetamines, heroin, and cocaine still account for a significant proportion of issues associated with illegal substance abuse. Reports from developed countries have revealed that NPS has been added to the existing classical illicit drugs.^[13]^ The emergence of NPS has had an additive effect rather than replacing classical illicit drugs, as it is cheaper than other stimulants.^[14]^

Polysubstance use is common among NPS users, including combinations of NPS and classical illicit drugs or a mixture of multiple NPS products.^[15]^ In Taiwan, a widely used illegal product called the “Toxic coffee packet” has been reported to be a mixture of various synthetic cathinones.^[16]^ The formula for such a mixture of NPS products changes rapidly, and there has been a recent increase in reports of substances in certain groups, such as synthetic opioids and hypnotics, according to the United Nations Office for Drugs and Crime.

The use of NPS poses challenges for emergency department (ED) physicians, as it is undetectable in simple urine toxicity tests. Identifying NPS use in patients heavily relies on clinical suspicion and judgment. The objective of our study is to report the clinical characteristics of patients who present to the emergency room in Taiwan after confirmed use of NPS and classical illicit substances. We compare the clinical characteristics of NPS users with those who combine NPS with classical illicit drugs to predict NPS use.

## 2. Methods

### 2.1. Study design and setting

We conducted a retrospective cohort study of patients who visited the ED from January 1st, 2019, to December 31st, 2019, with suspected classical substance use. The urine of the study participants was tested using liquid chromatography-mass spectrometry (LC-MS/MS) for qualitative analysis of 110 kinds of illicit drugs through the TEDAS project.^[17]^ The design and execution of this retrospective study were approved by the Institutional Review Boards of MacKay Memorial Hospital (20MMHIS036e) and the Poison Center, MacKay Memorial Hospital, Taipei, Taiwan.

### 2.2. Selection of participants

Patients were included if they underwent the NPS test in the ED. The criteria for performing the NPS test were as follows: patients who presented to the ED with altered mental status; patients with odd behavior, including abnormal eye movements and verbal or motor actions; patients who presented with a sympathomimetic toxidrome; patients who reported illicit substance use; patients with out-of-hospital cardiac arrest (OHCA) aged under 65 years; patients who attended the ED with a first seizure attack without an obvious cause; patients with a suicide attempt; patients with violent behavior; and patients with traumatic events and suspicion of illicit substance use. Patients with missing demographic data, such as age, sex, and final NPS results, were excluded from the study.

### 2.3. Variables

The general variables, such as age, sex, and the date of admission, were recorded. ED characteristics included initial vital signs, initial Glasgow Coma Scale at the ED, chief complaint, electrocardiography characteristics, laboratory results, brain computed tomography results, ED treatment, ED disposition, length of stay, and mortality at discharge.

### 2.4. Outcome

In the primary study, we divided the patients into 4 groups based on the NPS test results: NPS-positive and NPS-negative groups. In the secondary study, we classified patients into 2 groups: those who used NPS combined with classical illicit drugs (INPS group) and those with negative testing results.

### 2.5. Statistical analysis

The SPSS software (version 26.0; SPSS Inc., Armonk, NY) was used to calculate the baseline cohort characteristics, ED characteristics, and outcomes. We tested these characteristics using the chi-square test to compare categorical variables and independent t-tests to compare continuous variables. The threshold of statistical significance was set to a *P* value of <.05.

## 3. Results

**Figure 1.**
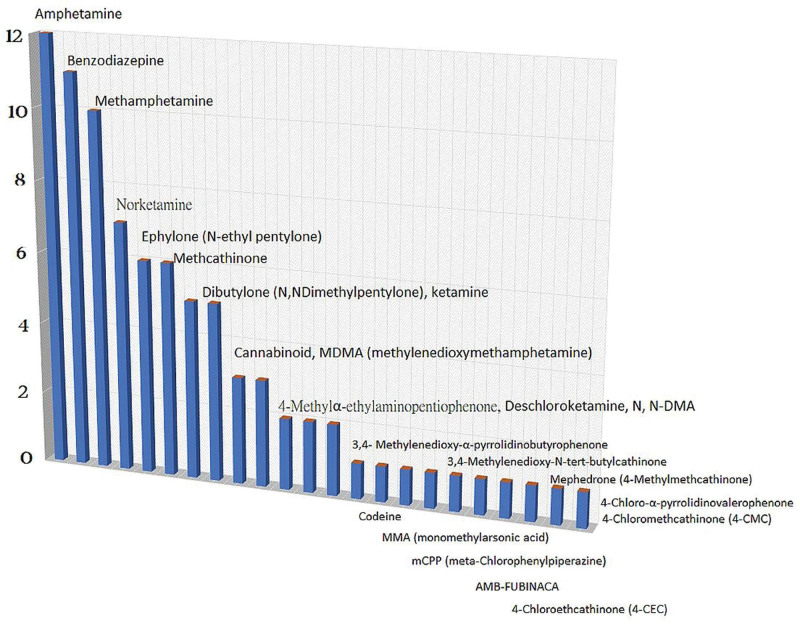
Eighty-four positive results of substances via 26 cases were found, of them, amphetamine (12, 14.3%) is the most common.

**Figure 2.**
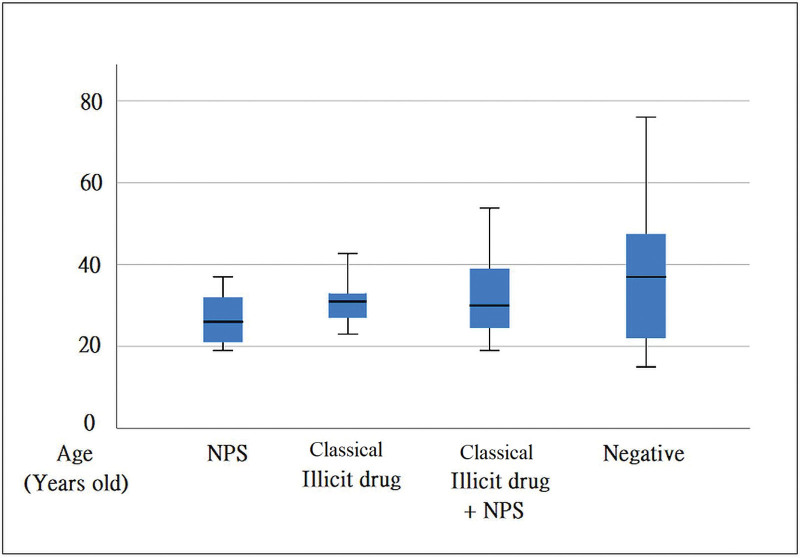
The NPS users are 11.1 years younger than negative result of toxic testing (26.4 vs 37.5, *P* = .005 < .05). NPS = new psychoactive substances.

**Figure 3.**
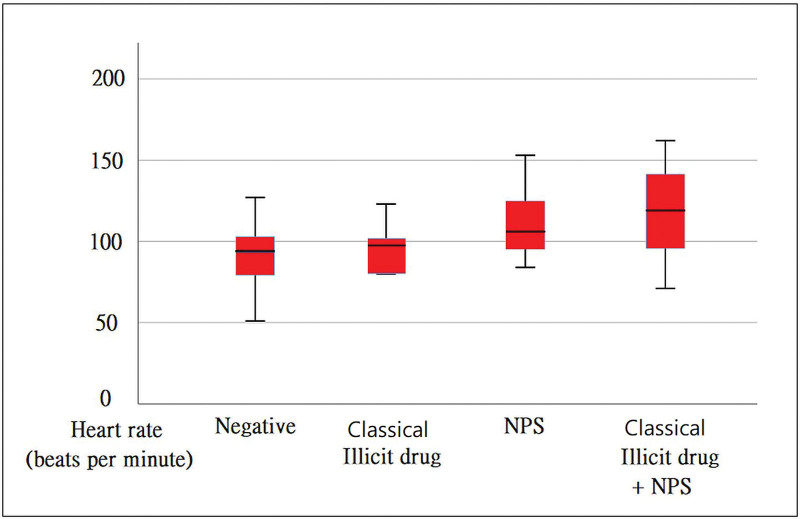
Cases in using illicit drug combined with NPS are tachycardic than negative results of toxic testing (119.6 vs 93.5, *P* = .024 < .05). NPS = new psychoactive substances.

Of the 58 patients enrolled, 11 were treated with NPS combined with a classical illicit drug (M:F = 6:5), and 9 were treated with NPS alone (M:F = 6:3). Six patients were classical illicit drug users (M:F = 6:3), and 32 tested negative for toxic substances. Positive results for 84 substances were found in 26 cases, with amphetamine (12, 14.3%) being the most common [Fig. 1]. Regardless of the type of NPS or classical illicit drug use, the majority of users were male. NPS users were, on average, 11.1 years younger than those who tested negative for drugs (26.4 vs 37.5, *P* = .005 < 0.05 [Table 1 and Fig. 2]). The heart rate of NPS users was 17.6 beats per minute faster than that of individuals with negative toxin screen results (111.1 vs 93.5, *P* = .046 < 0.05 [Fig. 3]).

**Table 1:** Comparisons in variable presentation and laboratory data between new psychoactive substance (NPS) use and negative result of toxic screening.

n, %	NPS (9, 22%)	Negative (32, 78%)	*P* value (two-tailed)
Age (yr old)	26.4 ± 6.4	37.5 ± 17.0	.005*
Gender (male: female)	6:3	19:13	.692
BT (degrees celsius)	37.0 ± 0.3	36.7 ± 1.0	.171
HR (/min)	111.1 ± 21.1	93.5 ± 21.6	.046*
RR (/min)	18.9 ± 0.9	18.7 ± 1.7	.633
SBP (mm Hg)	135.7 ± 15.9	125.8 ± 23.6	.160
DBP (mm Hg)	86 ± 12.8	75.8 ± 14.7	.062
GCS	12.9 ± 4.0	11.2 ± 4.0	.275
Eye opening	3.4 ± 1	3.2 ± 1.1	.125
Motor response	5.3 ± 1.7	4.8 ± 1.7	.372
Verbal response	4.1 ± 1.8	3.3 ± 1.7	.239
Oxygen saturatioin	100 ± 0	93.5 ± 6.2	.344
Bizarre behavior	(4, 44.4%)	(8, 25.0%)	.257
Delirium	(3, 33.3%)	(21, 65.6%)	.082
First time seizure	(2, 22.2%)	(1, 3.1%)	.052
Headache	(1, 11.1%)	(2, 6.3%)	.621
Palpitation	(1, 11.1%)	(1, 3.1%)	.326
Dyspnea	(0, 0%)	(3, 9.4%)	.340
Chest pain	(1, 11.1%)	(3, 9.4%)	.877
Nausea or vomiting	(3, 33.3%)	(8, 25%)	.618
Limb numbness/weakness	(1, 11.1%)	(3, 9.4%)	.877
Tremor	(1, 11.1%)	(2, 6.3%)	.621
ECG PR interval (ms)	140.1 ± 13.6	153.0 ± 28.5	.095
ECG QRS complex (ms)	89.7 ± 10.0	95.2 ± 14.0	.227
ECG QT_c_ (ms)	431.9 ± 33.3	459.9 ± 73.6	.151
WBC count	9444.4 ± 2534.8	10837.5 ± 4694.0	.251
platelet (micro-L)	324777.8 ± 794131.3	262156.3 ± 554315.1	.050*
Creatinine	0.9 ± 0.2	0.9 ± 0.4	.710
Sodium (meq/L)	139.1 ± 3.2	137.8 ± 6.2	.398
potassium (meq/L)	3.6 ± 0.5	3.5 ± 0.6	.594
CK (ng/mL)	424.8 ± 326.9	394.5 ± 411.0	.833
Ph	7.4 ± 0.0	7.3 ± 0.2	.255
P_a_CO^2^ (mm Hg)	42.1 ± 14.4	41.7 ± 14.6	.950
HCO_3_^−^ (mmol/L)	26.5 ± 3.7	22.9 ± 6.9	.100
intubated	(0, 0%)	(3, 9.4%)	.340
Use inotropic agent	(0, 0%)	(2, 6.3%)	.442
Hemodialysis	(0, 0%)	(1, 3.1%)	.591
LOS (d)	0.9 ± 0.3	0.5 ± 0.3	.554
Mortality	(0, 0%)	(2, 6.3%)	.442

BT = body temperature, CK = Creatine kinase, DBP = diastolic blood pressure, E = eye opening, ECG = electrocardiogram, GCS = Glasgow coma scale, GOT = Aspartate Aminotransferase, HR = heart rate, LOS = length of stay, M = motor response, QTc = corrected QT interval, RR = respiratory rate, SBP = systolic blood pressure, V = verbal response, WBC = white blood cell count.

* statistical significance.

The INPS group exhibited tachycardia compared to those who tested negative (119.6 vs 93.5, *P* = .024 < 0.05 [Table 2]). The incidence of palpitation symptoms was 8.8 times higher in the INPS group compared to those who tested negative for toxicity (27.3% vs 3.1%, *P* = .017 < 0.05).

**Table 2:** Comparisons in variable presentation and laboratory data between illicit drugs combined new psychoactive substance (NPS) use and negative group. INPS = patients who combined used illicit drug and NPS.

n, %	INPS (11, 25.6%)	Negative (32, 74.4%)	*P* value (two-tailed)
Age (yr old)	32.6 ± 10.4	37.5 ± 17.0	.269
Gender (male: female)	6:5	19:13	.779
BT (degrees Celsius)	37.1 ± 0.7	36.7 ± 1.0	.231
HR (/min)	119.6 ± 31.6	93.5 ± 21.6	.024*
RR (/min)	19.2 ± 1.3	18.7 ± 1.7	.329
SBP (mm Hg)	134.0 ± 20.2	125.8 ± 23.6	.280
DBP (mm Hg)	81.2 ± 17.6	75.8 ± 14.7	.381
GCS	11.1 ± 4.6	11.2 ± 4.0	.951
Eye opening	3.2 ± 1.3	3.2 ± 1.1	.990
Motor response	4.6 ± 1.9	4.8 ± 1.7	.864
Verbal response	3.3 ± 1.7	3.3 ± 1.7	.977
oxygen saturation	98 ± 0	93.5 ± 6.2	.048*
Bizarre behavior	(2, 18.2%)	(8, 25.0%)	.644
Delirium	(6, 54.5%)	(21, 65.6%)	.512
First time seizure	(1, 9.1%)	(1, 3.1%)	.163
Headache	(0, 0%)	(2, 6.3%)	.396
Palpitation	(3, 27.3%)	(1, 3.1%)	.017*
Dyspnea	(2, 18.2%)	(3, 9.4%)	.432
Chest pain	(1, 9.1%)	(3, 9.4%)	.978
Nausea or vomiting	(1, 9.1%)	(8, 25%)	.263
Limb numbness/weakness	(1, 9.1%)	(3, 9.4%)	.978
Tremor	(0, 0%)	(2, 6.3%)	.396
ECG PR interval (ms)	139.0 ± 24.6	153.0 ± 28.5	.329
ECG QRS complex (ms)	96.2 ± 11.4	95.2 ± 14.0	.830
ECG QT_c_ (ms)	460.1 ± 55.8	459.9 ± 76.3	.992
WBC count	10700.0 ± 4631.9	10837.5 ± 4694.0	.934
platelet	278454.5 ± 66981.1	262156.3 ± 554315.1	.479
Creatinine	1.0 ± 0.4	0.9 ± 0.4	.291
Sodium (meq/L)	140.0 ± 2.2	137.8 ± 6.2	.148
Potassium (meq/L)	3.5 ± 0.5	3.5 ± 0.6	.898
CK (ng/mL)	1731.1 ± 4525.5	394.5 ± 411.0	.432
Ph	7.4 ± 0.1	7.3 ± 0.2	.392
P_a_CO^2^ (mm Hg)	38.9 ± 7.2	41.7 ± 14.6	.503
HCO_3_^−^ (mmol/L)	23.0 ± 3.3	22.9 ± 6.9	.979
Intubated	(1, 9.1%)	(3, 9.4%)	.750
Use inotropic agent	(0, 0%)	(2, 6.3%)	.553
Hemodialysis	(0, 0%)	(1, 3.1%)	.553
LOS (d)	0.3 ± 0.2	0.5 ± 0.3	.170
Mortality	(0, 0%)	(2, 6.3%)	.396

BT = body temperature, CK = Creatine kinase, DBP = diastolic blood pressure, E = eye opening, ECG = electrocardiogram, GCS = Glasgow coma scale, GOT = Aspartate Aminotransferase, HR = heart rate, LOS = length of stay, M = motor response, QTc = corrected QT interval, RR = respiratory rate, SBP = systolic blood pressure, V = verbal response, WBC = white blood cell count.

* statistical significance.

In presentations by NPS users, we observed a synergistic effect of tachycardia in INPS patients. When comparing the basic characteristics, we found that the NPS group was associated with an increased heart rate (111.1 vs 93.5 beats/min, *P* < .05). Further investigation into NPS substance use in this group revealed that cathinone derivatives were predominantly consumed (9/9, 100%).

## 4. Discussion

The use of illicit drugs and overdose is a serious problem in the United States and Europe. The age-adjusted rate of overdose deaths was 207 per million in 2018 in the United States and 23.7 per million in Europe.^[18]^ However, in Northeast Asia, synthetic cannabinoids, cathinones, and phenethylamines are the most commonly detected NPS.^[19]^ Overdose from synthetic cannabinoids and cathinones can cause agitation, psychosis, confusion, seizures, hypertension, tachycardia, chest pain, and nausea. In this study, we identified several characteristics of NPS abuse and the combined abuse of NPS and classical illicit drugs.

### 4.1. NPS users were found to be on average 11.1 years younger than individuals who tested negative for toxic substances when visiting the ED

Regarding the analysis of age and gender, this study revealed that patients confirmed to have used NPS were significantly younger, with a mean age of 26.4 years, compared to those who tested negative (mean age: 37.5 years, *P* < .05). This finding aligns with a previous global review that indicated younger individuals have easier access to NPS.^[9]^ The review also highlighted the use of specific marketing strategies employed by drug dealers to target younger demographics, such as advertising through social media, offering free samples, and using colorful packaging.^[9]^ Another study conducted in Australia, which examined deaths related to NPS, reported a mean age of 30.7 years.^[20]^ These findings underscore the public health concern of NPS prevalence among younger populations. In terms of sex differences, male NPS users exhibit more aggressive behavior, higher rates of concurrent human immunodeficiency virus infection, rhabdomyolysis, and greater likelihood of admission to the intensive care unit, compared to female users.^[21]^ Interestingly, the use of NPS appears to be more frequent among young individuals in psychiatric populations, while alcohol consumption is more prevalent in healthy young populations.^[22]^

### 4.2. NPS increases heart rate by 26.1 beats per minute compared to individuals who test negative for toxic substances in the ED

In our study, we observed that the heart rate of NPS users was 17.6 beats per minute faster than that of individuals who tested negative for toxicity. Synthetic cathinones, which are structurally similar to amphetamines, exhibit similar cardiovascular effects and complications, including tachycardia, myocardial infarction, and stroke.^[23]^ Furthermore, the INPS group, which comprised individuals using a combination of cathinone and ketamine, demonstrated an even higher heart rate (119.6 vs 93.5 beats, *P* < .05). Previous research has reported that ketamine can cause tachycardia.^[24]^ The observed higher heart rate in the INPS group could potentially be attributed to the combined use of cathinone and ketamine. However, the possibility of a synergistic effect between cathinone and ketamine has not yet been reported.

### 4.3. Platelet count of NPS users is higher than other people visiting ED who are negative to toxic screen

Although the platelet count in NPS users and individuals with negative toxic testing results falls within the normal range of 150,000 to 450,000 per μL, the platelet count of NPS users is slightly higher than that of those with negative results (324,777 vs 262,156, *P* = .05). No previous reports have described the relationship between platelets and NPS. Some NPS, such as synthetic cannabinoid compounds, have been associated with serious physical consequences like myocardial infarction, seizures, and renal damage.^[25]^ This raises concerns about whether the slight increase in platelet count in NPS users contributes to blood hyperviscosity, potentially leading to myocardial infarction.

### 4.4. Relative 4.5% higher pulse oximeter value INPS group than other people visiting ED who are negative to toxic screen

In the emergency room, various manifestations raise suspicion of illicit drug or NPS use, including suicide events, bizarre behavior and speech, mood changes, altered perception, thinking, memory, attention, agitation, panic, dysphoria, OHCA, seizures, acute psychosis, delirium, unconsciousness, violence, falls or jumping from tall buildings, traffic accidents, and acute vascular events.^[13,20,26–31]^ The combined use of classical illicit drugs and NPS can mimic severe or debilitating conditions affecting the heart, liver, lungs, and kidneys, leading to altered consciousness, delirium, psychosis, seizures, accidents, and vascular events. However, patients with negative toxicity test results have a slightly lower arterial oxygen saturation (4.5% lower) compared to those using classical illicit drugs combined with NPS (93.5 vs 98, *P* = .048 < .05). This suggests that patients using classical illicit drugs combined with NPS have better respiratory function than those who test negative for toxic substances.

### 4.5. NPS combined classical illicit drugs users have 8.8-fold palpitation sensation than that of other people visiting ED who are negative to toxic screen

Palpitation is a symptom commonly experienced in both classical illicit drug use and NPS use.^[16]^ Patients in the INPS group, who used classical illicit drugs combined with NPS, exhibited a significantly higher occurrence (8.8-fold) of palpitations compared to those with negative toxic testing results. However, there was no statistically significant difference between the INPS group and the negative testing group in other symptoms such as bizarre behavior, delirium, first-time seizure, headache, dyspnea, chest pain, nausea and vomiting, limb numbness, and tremor. Therefore, these symptoms alone cannot clearly distinguish between INPS users and patients with negative toxic testing results, except for palpitation. In the treatment of NPS poisoning, it is crucial to prioritize stabilizing the airway, breathing, and circulation, maintaining normal body temperature, managing agitation, and addressing dehydration and rhabdomyolysis.^[32]^ According to a multicenter study conducted in 2022, the overall mortality rate among NPS users is approximately 4.5%.^[17]^

## 5. Conclusion

Bizarre behavior and speech, changes in mental state, memory, attention, feelings of agitation, panic, dysphoria, seizures, acute psychosis, delirium, unconsciousness, OHCA, trauma, and acute vascular events are commonly observed in the ED. These symptoms and presentations can also be seen in several debilitated patients in internal, surgical, and neurological departments. In our study, we found that younger age, tachycardia, palpitations, and relatively normal oxygen saturation are indicators that raise suspicion of NPS use. If the ED staff is unable to differentiate NPS use from other debilitating conditions or if there is a suspicion, it is advisable to contact the local poison center for further assessment, particularly for urine toxicity testing.

## 6. Limitations

Because this study is conducted at a single center, a relatively small number of cases were enrolled, which led to some statistical ambiguity, such as the difference in platelet counts between NPS users and patients with negative results of toxic testing. We anticipate that this distinction will become more pronounced and clearly differentiated with a larger number of participants.

## Acknowledgments

We thank the Taiwan Food and Drug Administration, Taiwan Society of Emergency Medicine, and MacKay Memorial Hospital.

## Author contributions

**Conceptualization:** Yu-Jang Su, Kuo-Song Chang.

**Data curation:** Yu-Jang Su, Tse-Hao Chen.

**Formal analysis:** Yu-Jang Su.

**Investigation:** Yu-Jang Su, Kuo-Song Chang, Yen-Chun Lai.

**Methodology:** Yu-Jang Su.

**Project administration:** Yu-Jang Su.

**Software:** Yu-Jang Su.

**Supervision:** Yu-Jang Su.

**Validation:** Yu-Jang Su.

**Writing – original draft:** Yu-Jang Su, Tse-Hao Chen, Wei-Hsiang Liao, Yen-Chun Lai.

**Writing – review & editing:** Yu-Jang Su.
